# Supplementary material for: Canada Goldenrod Invasion Regulates the Effects of Soil Moisture on Soil Respiration
Source: Int J Environ Res Public Health. 2022 Nov 22;19(23):15446. doi: 10.3390/ijerph192315446 (PMC9741181; doi:10.3390/ijerph192315446)
Supplement: Supplementary file 1 [file ijerph-19-15446-s001.zip › ijerph-1986266-Table S1.pdf]

Table S1. The variations (Δ) of soil characteristics between start-time sampling (X_{t1}) and end time sampling (X_{t2}) under different treatments ($\Delta = X_{t2} - X_{t1}$).

	Low water level			Intermediate water level			High water level			Treatment effect
	NI	II	CI	NI	II	CI	NI	II	CI	
pH	-0.41±0.04	-0.43±0.06	-0.35±0.22	-0.60±0.10	-0.58±0.08	-0.52±0.12	-0.60±0.17	-0.60±0.12	-0.59±0.18	WL**
NO_3^- (×10^{-3} mg g^{-1} soil)	-2.85±3.73	-0.23±0.97	-6.04±6.58	-2.77±1.45	-3.56±5.67	-7.87±0.33	3.03±3.80	1.51±1.21	1.81±0.45	W**
DOC (×10^{-1} mg C g^{-1} soil)	0.84±0.05	1.0±0.32	1.3±0.47	0.66±0.22	0.66±0.55	0.83±0.63	0.30±0.14	0.40±0.18	0.48±0.29	WL**
DON (×10^{-3} mg C g^{-1} soil)	-11.62±17.97	-9.40±15.00	-13.62±10.94	-2.03±4.31	-7.50±2.67	-12.08±4.68	0.58±2.89	0.56±1.77	1.81±0.83	WL**
SAP (×10^{-2} mg P g^{-1} soil)	-0.20±0.10	-0.08±0.14	-0.25±0.04	-2.31±1.98	-2.09±1.88	-1.21±2.05	1.03±0.17	0.87±0.18	1.07±0.23	WL**
STC (×10^{-1}mg C g^{-1} soil)	-0.78±2.44	-3.99±4.58	-5.82±1.83	-0.84±7.14	-2.23±6.90	2.36±5.94	0.15±2.15	0.86±6.30	-0.77±3.99	
STN (mg N g^{-1} soil)	0.96±2.39	1.06±2.24	1.79±2.54	-1.06±0.27	-1.62±0.23	-1.11±0.23	-1.23±0.81	-1.42±0.348	-0.95±0.49	WL**
STP (×10^{-2} mg P g^{-1} soil)	3.16±2.29	-1.44±8.77	-1.02±9.31	-1.54±1.61	-2.07±3.11	-1.84±1.17	-2.54±2.93	-3.49±3.98	1.93±2.01	
$ST_{C:P}$	-0.37±0.53	-0.45±0.21	-0.76±0.80	0.26±0.97	-0.15±0.84	0.50±0.77	0.27±0.56	0.42±1.03	-0.28±0.77	WL*
DOC:STC (×10^{-2})	2.29±0.10	3.09±1.16	4.01±1.25	1.72±0.21	1.96±1.40	2.16±1.38	0.81±0.57	1.04±0.29	1.40±1.10	WL**
MBC (mg C g^{-1} soil)	2.27±0.19	2.08±0.11	2.01±0.16	2.15±1.14	2.04±0.46	1.77±0.34	1.90±0.49	1.91±0.34	1.88±0.55	
MBN (×10^{-3} mg N g^{-1} soil)	-5.90±0.77	-6.00±1.15	-5.11±1.35	-4.34±3.76	-4.99±2.54	-7.56±2.46	-6.98±1.11	-5.65±0.59	-6.83±2.03	

MBP ($\times10^{-3}$ mg P g^{-1} soil)	-0.36±1.78	0.46±3.25	-1.04±2.40	4.62±0.31	-0.18±1.65	-1.25±3.02	-1.98±1.77	-2.05±0.70	0.92±2.07	
$MB_{C:N}$ ($\times10^{3}$)	1.90±0.54	1.30±0.86	1.22±0.82	1.59±1.99	1.15±0.18	1.65±0.91	2.13±1.12	1.93±0.99	3.68±4.20	
$MB_{C:P}$ ($\times10^{2}$)	9.55±5.52	3.85±0.68	7.69±5.99	2.41±1.18	7.25±7.20	3.28±0.73	4.89±2.47	7.85±7.80	8.95±10.51	
H′	-0.63±0.51	-0.28±0.54	0.48±0.65	-1.05±0.42	-2.30±0.74	-0.73±1.08	-1.55±1.20	-1.87±1.05	-0.29±0.69	SI*; WL**; SI×WL
EEA_{C} (μg h^{-1} g^{-1} soil)	-0.03±1.98	0.51±1.02	4.74±9.74	0.49±1.10	-1.83±2.11	1.55±2.19	-0.33±3.63	0.32±0.93	1.69±1.97	
EEA_{N} ($\times10^{2}$μg h^{-1} g^{-1} soil)	-0.91±0.41	-1.02±0.24	-0.87±0.27	-1.62±1.50	-0.96±0.50	-0.88±0.73	-0.65±0.30	-0.36±0.19	-0.44±0.50	WL**
EEA_{P} (μg h^{-1} g^{-1} soil)	-5.42±3.07	-2.47±1.69	4.26±5.28	0.64±3.64	6.54±5.93	8.92±8.46	-2.38±11.20	-1.97±8.83	-6.32±10.65	WL**
$EEA_{C:P}$	0.76±1.02	0.27±0.05	0.01±0.75	-0.43±0.95	-9.68±15.18	-0.90±0.78	-1.65±6.36	-3.15±6.31	-0.51±1.60	
VL	0.81±1.00	0.41±0.02	0.63±1.31	-0.11±0.55	-9.41±15.28	-0.60±0.86	-0.40±4.69	-2.96±6.23	-0.43±1.60	
VA ($\times10^{1}$)	1.39±1.22	3.29±0.61	5.67±2.08	3.71±2.42	6.54±1.00	5.07±1.28	3.19±2.49	2.57±2.02	0.47±0.12	WL**; SI×WL
CUE ($\times10^{-1}$)	-1.07±1.34	-1.56±0.57	-2.99±0.80	-0.77±0.81	-1.88±1.57	-1.64±1.09	0.26±0.65	±-0.16±0.80	-0.29±0.85	SI*; WL**

SI = *Solidago canadensis* L. invasion, WL =water level treatment, SI×WL = interactive effect of SI and WL, NI = non-invasive stage treatment, II = intermediate invasive treatment, CI = completely invasive stage treatment. $N0_3^-$ = nitrate nitrogen, DOC = dissolved organic carbon,DON = dissolved organic nitrogen, SAP = soil available phosphorus, STC = soil total carbon, STN = soil total nitrogen, STP = soil total phosphorus, $ST_{C:P}$ = the ratio of STC to STP, DOC:STC = the ratio of DOC to STC, MBC = micorbial biomass carbon, MBN = micorbial biomass nitrogen, MBP = micorbial biomass phosphorus, $MB_{C:P}$ = the ratio of MBC to MBP, H' = soil microbial community diversity, EEA_{C} = extracellular carbon-acquiring enzymic activity, EEA_{N} = extracellular nitrogen-acquiring enzymic activity, EEA_{P} = extracellular phosphorus-acquiring enzymic activity, $EEA_{C:N}$ = the ratio of EEA_{C} to EEA_{N}, $EEA_{C:P}$ = the ratio of EEA_{C} to EEA_{P}, VL = microbial energy limitation VA = microbial nutrient limitation, CUE = microbial carbon utilization. * and ** present significant at $P < 0.05$ and $P < 0.01$, respectively.
