# Supplementary figures and images for: Canada Goldenrod Invasion Regulates the Effects of Soil Moisture on Soil Respiration
Source: Int J Environ Res Public Health. 2022 Nov 22;19(23):15446. doi: 10.3390/ijerph192315446 (PMC9741181; doi:10.3390/ijerph192315446)

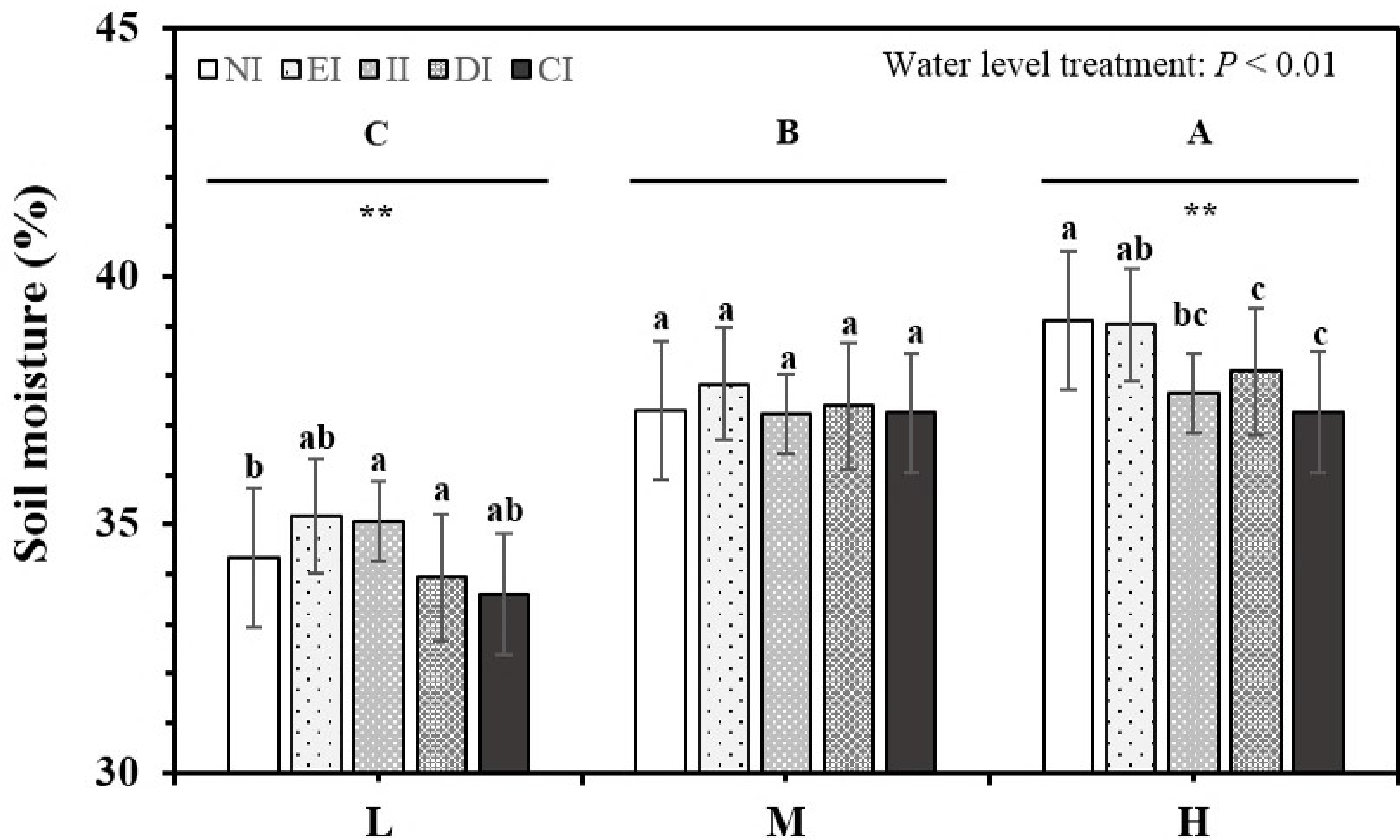

Supplement: Supplementary file 1 [file ijerph-19-15446-s001.zip › ijerph-1986266-Figure S1.pdf]
